# New QTLs for Spot Blotch Disease Resistance in Wheat (*Triticum aestivum* L.) Using Genome-Wide Association Mapping

**DOI:** 10.3389/fgene.2020.613217

**Published:** 2021-01-14

**Authors:** Vipin Tomar, Daljit Singh, Guriqbal Singh Dhillon, Ravi Prakash Singh, Jesse Poland, Arun Kumar Joshi, Pawan Kumar Singh, Pradeep Kumar Bhati, Suneel Kumar, Mokhlesur Rahman, Budhi Sagar Tiwari, Uttam Kumar

**Affiliations:** ^1^Borlaug Institute for South Asia, Ludhiana, India; ^2^Institute of Advanced Research, Gandhinagar, Gujarat, India; ^3^The Climate Corporation, Bayer Crop Science, Creve Coeur, MO, United States; ^4^Department of Biotechnology, Thapar Institute of Engineering and Technology, Patiala, India; ^5^Global Wheat Program, International Maize and Wheat Improvement Center (CIMMYT), Texcoco, Mexico; ^6^Department of Plant Pathology, Kansas State University, Manhattan, KS, United States; ^7^International Maize and Wheat Improvement Centre, New Delhi, India; ^8^Wheat Research Center, Regional Agricultural Research Station, Bangladesh Agricultural Research Institute, Jamalpur, Bangladesh

**Keywords:** GWAS, GBS, spot blotch, QTLs, BLUPs, wheat

## Abstract

Spot blotch disease caused by *Bipolaris sorokiniana* is a major constraint for wheat production in tropics and subtropics. The introgression of spot blotch resistance alleles to the disease susceptible lines is critical to securing the wheat production in these regions. Although genome-wide association studies (GWASs) for spot blotch were attempted earlier, the present study focused on identifying new quantitative trait loci (QTLs) for spot blotch under natural disease pressure in diverse field conditions. A total of 139 advanced spring wheat lines were evaluated in three environments (three years and two locations) in India and Bangladesh. The GWAS using 14,063 polymorphic genotyping-by-sequencing (GBS) markers identified eight QTLs associated with spot blotch disease resistance belonging to eight chromosomes across the wheat genome. Here, we report the identified marker–trait associations (MTAs), along with the allele effects associated with the disease. The functional annotation of the significant markers identified NBS-LRR, MADS-box transcription factor, and 34 other plant-related protein families across multiple chromosomal regions. The results indicate four promising new QTLs on chromosomes 1A (497.2 Mb), 1D (89.84 Mb), 2B (421.92 Mb), and 6D (6.84 Mb) associated with several disease resistance protein families. These results provide insights into new genomic regions associated with spot blotch disease, and with additional validation, could be utilized in disease resistance breeding efforts in wheat development.

## Introduction

Wheat (*Triticum aestivum* L.) is the primary staple food for more than 35% of the world’s population (FAO, 2018). The pace of wheat improvement must accelerate to meet the projected global food demand by 2050. The Green Revolution played a crucial role in India, Pakistan, Nepal, and Bangladesh, ensuring food security in this densely populated region of the world (Joshi et al., 2007b). However, wheat production faces multiple threats via rapidly evolving pathogen variants, pests, and increased climate variability, which considerably jeopardizes crop productivity (Sharma et al., 2007b; Friesen et al., 2008; Gurung et al., 2009, 2012). Breeding wheat for climatic resilience and disease resistance combined with good agronomic value can potentially improve wheat productivity to meet future food demands (Mondal et al., 2016).

Spot blotch caused by *Bipolaris sorokiniana* is a major constraint in wheat production in tropics and subtropics (Dubin and Van Ginkel, 1991; van Ginkel and Rajaram, 1998). The pathogen has a worldwide dispersal, but it is predominantly aggressive under warm, high relative humidity, and high temperature associated conditions with imbalanced soil fertility. Major yield losses are observed in the fields with lower inputs and under late-sown conditions (Joshi et al., 2007b). *B. sorokiniana* acts as a causal agent for numerous diseases like head blight, seedling blight, foliar blight/spot blotch, common root rot, and black point of wheat, barley, other small cereal grains and grasses (Lennard, 1984). Therefore, spot blotch of wheat is considered as one of the most crucial diseases caused by this pathogen in the mega-environments characterized by temperature above 17°C and high humidity (van Ginkel and Rajaram, 1998).

It is often difficult to achieve the desired level of host resistance to several diseases through conventional breeding. In the case of wheat disease, resistance is inherited both qualitatively and quantitatively (Kumar et al., 2009, 2010; Marone et al., 2013; French et al., 2016). The genetic basis of spot blotch resistance has earlier been documented as eight major quantitative trait loci (QTLs), namely, *QSb.bhu-2A*, *QSb.bhu-2B*, *QSb.bhu-2D*, *QSb.bhu-3B*, *QSb.bhu-5B*, *QSb.bhu-6D*, *QSb.bhu-7B*, and *QSb.bhu-7D* (Kumar et al., 2009, 2010). Sharma et al. (2007a) reported three microsatellite markers (*Xgwm67*, *Xgwm570*, and *Xgwm469*) linked with spot blotch resistance. The QTL *QSb.bhu-5B*, *QSb.bhu-7D*, and *QSb.bhu-3B* have been designated as genes *Sb1*, *Sb2*, and *Sb3*, respectively, in further studies (Lillemo et al., 2013; Kumar et al., 2015; Lu et al., 2016). *Lr34* and *Lr46*, the two broadly used genes conferring leaf rust resistance, have also been reported to contribute to spot blotch resistance. While the *Lr34* gene has been reported to be the major locus on chromosome 7D explaining up to 55% phenotypic variation for spot blotch disease resistance, this locus was given the gene designation *Sb1* (Lillemo et al., 2013). Nevertheless, in the past few years, several QTLs and genetic markers for spot blotch resistance have been identified in multiple studies in wheat (Gurung et al., 2014; Zhu et al., 2014; Singh et al., 2018).

Due to evolutionary changes in pathogen populations, resistance genes may lose their effectiveness over time. Given these challenges, identification and mapping of novel resistance genes would aid breeding for disease resistance in wheat. One of the approaches is to identify spot blotch resistance QTLs through association mapping. This approach leverages historical recombination and generally has better mapping resolution compared to biparental mapping (Zhu et al., 2008). The genome-wide association study (GWAS) using genotyping by sequencing (GBS) to identify QTLs for traits of interest provides advantage through better genome coverage compared to conventional marker systems like SSRs, AFLP, and CAPs. The GBS utilizes the advantage of high-throughput genotyping assays with relatively low data costs, which are much higher in genome sequencing and re-sequencing techniques (Elshire et al., 2011). A fundamental approach in GWAS is to have enough genome coverage so that functional alleles will be in linkage disequilibrium (LD) with at least one marker (Myles et al., 2009). The association studies for disease resistance, including spot blotch, have been reported in some of earlier studies (Maccaferri et al., 2010, 2015; Yu et al., 2011; Zegeye et al., 2014; Ayana et al., 2018).

There are limited reports where the same set of genotypes is exposed to natural disease pressure to identify genomic regions underpinning wheat spot blotch resistance. Thus, the main objective of this study was to establish marker–trait associations (MTAs) for spot blotch resistance using GBS markers in spring wheat specific to the South Asian regions, *viz*., India and Bangladesh, the well-known hot spots for this disease. We also aimed to identify novel QTLs and validated known genomic loci conferring spot blotch resistance in wheat.

## Materials and Methods

### Plant Material and Field Layout

The population was a collection of 139 advanced breeding lines of spring wheat (Supplementary Table S1) derived from the crosses where elite high-yield breeding lines were used as parents. The advanced lines were sent to South Asia as part of the CIMMYT wheat breeding program aiming to develop high-yielding varieties suitable for South Asia. They were carefully assembled to avoid the confounding effects of phenology after multiyear and multilocation trials. The lines were evaluated in two replicates at two field locations: Bangladesh Agricultural Research Institute (BARI), Jamalpur, Bangladesh (24°22′07.7″N, 88°39′42.0″E), during 2017 and Borlaug Institute for South Asia (BISA), Pusa (25°57′22.8″N, 85°40′13.1″E), in north India during 2019 and 2020 seasons in a randomized block design. For convenience, the different location–season combinations were termed as Env1 (Pusa19), Env2 (Pusa20), and Env3 (BARI17). Pusa, India, and Jamalpur, Bangladesh, are known hot spots for spot blotch disease (Sharma et al., 2007b). The trials were timely sown with full irrigation applied through gravity flood irrigation. The spreader rows of susceptible variety Sonalika were also planted for creating epiphytotic disease conditions. Besides, four auxiliary gravity flood irrigations were also given at regular intervals. All agronomic practices like fertilization and weeding were performed as recommended for each location.

### Screening for Spot Blotch Disease Resistance

The material was evaluated under natural infection conditions in the field. Spot blotch response was evaluated thrice during the mid to advanced phases of disease development, i.e., between heading (Growth Stage 50 or GS50 on Zadoks scale) and grain filling stage (GS80) (Zadoks et al., 1974). The disease severity (SEV) was recorded visually on a 0–100 scale where 0 is complete resistance and 100 is completely susceptible.

### Genotyping

Seeds of all lines were obtained from the CIMMYT genetic resource program, and genomic DNA was extracted from five bulked leaves using a modified CTAB procedure as described in Dreisigacker et al. (2016) in CIMMYT, Mexico. The DNA samples were sent to Kansas State University, United States, for GBS, described by Poland et al. (2012) and sequenced with Illumina HISeq2500. GBS-SNP markers were called with TASSEL *v*5.2 pipeline GBSv2 (Bradbury et al., 2007) and aligned to the reference Chinese Spring Wheat Assembly (RefSeq v1.0). The following SNP filtering criteria were applied on raw SNP calls: less than 30% missing markers, minimum 5% minor allele frequency (MAF), and less than 20% heterozygosity. The filtering step yielded 14,063 markers, and the remaining missing values were imputed using Beagle v4.1 (Browning and Browning, 2016).

### Statistical Analysis

The experimental design in each environment was a randomized complete block design with two replications per environment/location. The best linear unbiased prediction (BLUP) values were obtained through META-R v6.03 (Alvarado et al., 2020), developed by CIMMYT, Mexico, using the following formula:

$$Y_{\mathrm{ik}}=\mu +{\mathrm{Rep}}_{i}+{\mathrm{Gen}}_{k}+\epsilon_{\mathrm{ik}}\,\left(\mathrm{within}\,\mathrm{the}\,\mathrm{environment}\right)$$

$$Y_{\mathrm{ijk}}=\mu +{\mathrm{Rep}}_{i}\,\left({\mathrm{Env}}_{j}\right)+{\mathrm{Env}}_{j}\times {\mathrm{Gen}}_{k}+{\mathrm{Gen}}_{k}+{\mathrm{Env}}_{j}+\epsilon_{\mathrm{ijk}}\left(\mathrm{across}\,\mathrm{environments}\right)$$

where *Y*_*ik*_ is the trait of interest, μ is the mean effect, *Rep*_*i*_ is the effect of the *i*th replicate, *Gen*_*k*_ is the effect of the *k*th genotype, *ϵ*_*ik*_ is the error associated with the *i*th replication, and the *k*th genotype, which is assumed to be normally and independently distributed, with mean zero and homoscedastic variance σ^2^. For across environments, *Y*_*ijk*_ is the trait response,*Env*_*j*_ is the *j*th environment, *Rep*_*i*_(*Env*_*j*_) is the effect of *i*th replication in the *j*th environment, and *Env*_*j*_×*Gen*_*k*_ is the environment and genotype interaction. The resulting analysis produced the adjusted trait phenotypic values in the form of BLUP within and across environments. The BLUP model considers genotypes as random effects, minimizing the effect of screening time and other environmental effects on the spot blotch severity.

Besides, the components of the phenotypic variance of a given trait at an individual environment were also extracted to calculate broad-sense heritability using the formula as:

$$h_{2}=\frac{\sigma_{g}^{2}}{\sigma_{g}^{2}+\sigma_{e}^{2}/n\,\mathrm{reps}}\,\left(\mathrm{within}\,\mathrm{the}\,\mathrm{environment}\right)$$

$$h^{2}=\frac{\sigma_{g}^{2}}{\sigma_{g}^{2}+\sigma_{\mathrm{ge}}^{2}/n\,\mathrm{env}+\sigma_{e}^{2}/\left(n\,\mathrm{reps}\times n\,\mathrm{env}\right)}\left(\mathrm{across}\,\mathrm{environments}\right)$$

where $\sigma_{g}^{2}$ and $\sigma_{e}^{2}$ are the genotype and error variance components, respectively, $\sigma_{g\,e}^{2}$ is the genotype by environment interaction variance, *n env* is the number of environments, and *n reps* is the number of replicates. All effects are considered random for calculating the BLUP and broad-sense heritability. The BLUP phenotypic distributions of disease scores at each environment were plotted to check normality assumptions.

### Principal Component Analysis and Linkage Disequilibrium

Principal component analysis (PCA) was performed using 14,063 SNPs and 139 genotypes in FarmCPU (Liu et al., 2016). The first two principal components were drawn to show the clustering among genotypes. The population structure (PC) and LD were estimated in TASSELv5.2 (Bradbury et al., 2007). The intra-chromosomal LD was calculated as the pairwise marker correlations (*r*^2^) between the SNP markers plotted against the physical distance for significant MTAs. The long-distance LD and spline were fitted to the LD-decay graph using *r*^2^ values of less than 0.99 using ggplot2 v3.30 in R v3.5.2 (R Core Team, 2019).

### Genome-Wide Association Analysis

The FarmCPU (Liu et al., 2016) model of GAPIT 3.0 was used to test the MTA between the SNP markers and spot blotch disease severity (SEV) and to take advantage of the mixed linear model (MLM) and stepwise regression [fixed-effect model (FEM)]. This algorithm uses both the FEM and the random-effect model (REM) iteratively where FEM is employed to test *m* genetic markers, and associations or pseudo-quantitative trait nucleotides are included as covariates to control false positives in REM.

Subsequent GWAS analysis was performed using 14,063 SNPs scored on 139 lines with phenotypic data of disease score from the seasons 2017, 2019, and 2020. Given the exploratory nature of this study, we used a relatively less-stringent *p-value* threshold of 0.003 (–log_10_*P* = 2.5) to avoid removing true positive associations. To uncover the stable disease resistance QTLs, association signals that were significant across two or more environments were selected. The allelic effects were further investigated to identify significantly associated markers in lieu of phenotypic data for studying the importance of individual alleles in spot blotch disease resistance.

### Gene Functional Annotations

Genome-wide association study results were further analyzed to test if the MTAs fall within the known genic regions using functional annotation from the reference genome assembly (IWGSC Ref Seq v1.0). Functional annotation of the genes either harboring significant SNPs or adjacent to the SNPs was retrieved and examined for their association with spot blotch resistance from the genome annotations provided by IWGSC. Subsequently, protein functions were literature mined from annotated information.

### Physical Mapping

From the physical map prepared, we identified the most significant QTLs found on eight wheat chromosomes. A total of 29 SNPs were mapped and used from GWAS wherein a group was considered to be different if physical distance is more than 5 Mb. New QTLs of the present study and genes/markers associated with SB resistance from previous studies were plotted for physical mapping.

## Results

### Estimation of Heritability

**FIGURE 1:**
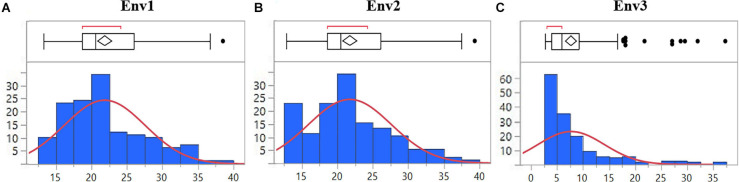
Distribution of 139 advanced wheat breeding lines for spot blotch severity (%) based on BLUPs in **(A)** Env1, **(B)** Env2, and **(C)** Env3 environments. The lines in the boxplot represent the median of the distribution and the diamond represents the model disease score, while the black dots are outliers.

The mean disease severity of the population ranged from 2.65 to 39.35 in three environments (2017, 2019, and 2020), including Pusa, India, and BARI, Bangladesh (Supplementary Table S2). The highest mean disease severity was recorded in Pusa20 (Env2) while the lowest was at BARI17 (Env3). The analysis of variance revealed the highest heritability in Env3 (0.82) and the lowest in Env1 (0.72). Based on the combined analysis of all environments, we observed high heritability (0.76). There were significant Genotype × Environment interactions (*P* < 0.0001; Table 1). The populations displayed significant phenotypic variation for spot blotch resistance with a nearly continuous distribution of lines in all environments (Figure 1).

**TABLE 1:** Analysis of variance of 139 advanced lines evaluated for spot blotch disease resistance in four environments based on BLUP of disease severity recorded at GS 77 on Zadoks scale at BISA Pusa (India) and BARI, Jamalpur (Bangladesh).

**Statistics**	**Env1**	**Env2**	**Env3**	**Combined**
Heritability	0.72	0.76	0.82	0.76
Genotype variance	45.12	45.04	49.80	31.21
Residual variance	35.12	28.95	22.60	28.89
Grand mean	21.88	21.78	7.72	17.13
LSD	7.04	6.56	6.00	05.42
CV	27.09	24.70	61.59	31.37
N replicates	2	2	2	2
Genotype significance	4.58E-13	1.44E-15	0	1.55E-23
Gen × Env significance	−	−	−	2.30E-11
Gen × Loc variance	−	−	−	15.43

*LSD, least significant difference; CV, coefficient of variation; N replicates, number of replicates; Env, environment.*

### SNP Density and Principal Component Analysis

**FIGURE 2:**
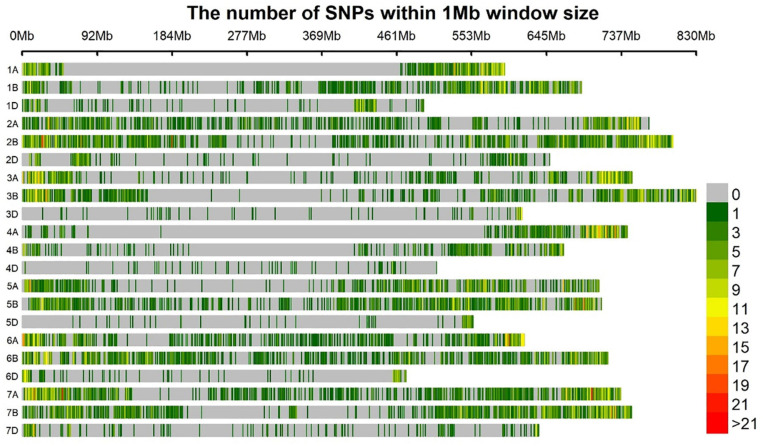
Bar plot showing the densities of 14,063 GBS markers on the 21 wheat chromosomes. The color key with marker densities indicates the number of markers within a window size of 1 Mb.

Among polymorphic SNP markers, 40.9% (5754), 50.8% (7142), and 8.3% (1167) were from the A, B, and D genomes, respectively. With a genomic coverage of 13.9 GB and 14,063 markers across the genome, the average marker density was 1.9 Mb per marker. The lowest marker density 7.03 Mb per marker was at chromosome 4D while the highest 0.54 Mb per marker was observed at chromosome 2B. The average distance between markers for A, B, and D genomes was 0.89, 0.84, and 3.92 Mb, respectively (Figure 2).

**FIGURE 3:**
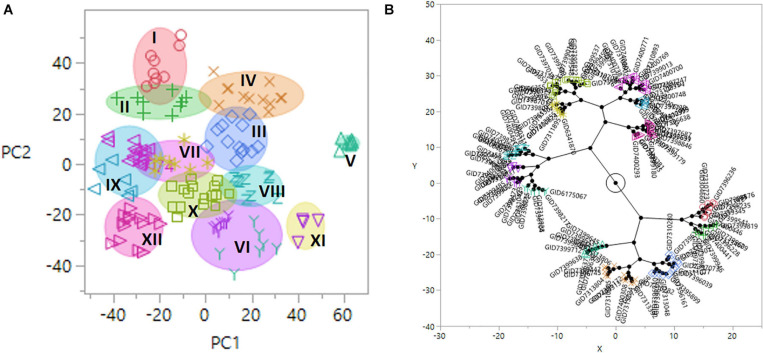
**(A)** Diagram of principal component (PC) 1 and 2 vectors calculated by the principal component analysis (PCA) of 14,063 genotyping-by-sequencing (GBS) single-nucleotide polymorphism (SNP) markers in 139 advanced wheat breeding lines, plotted in 12 population groups (I–XII). The *x*-axis and *y*-axis represent projections of the PC1 and PC2, respectively. **(B)** Constellation plot using the Ward method in JMP *v.*14.

Population structure was determined using PCA, where genotypes were clustered into 12 groups using the Ward method in JMP v.14 (Figure 3). Group 1 (G–I) consisted of nine lines, including the resistant check HD2733. Group G–X consisted of a maximum of 16 lines, while the minimum number of lines is in the group G–XI (six lines). Based on pedigree information, most of the lines in a group shared allele descended from common parents. The lines without common parents or less than three sibs per family were classified as group G-XII. The largest group (G–X) consisted of lines with mixed pedigrees, including TEPOKA, TRCH, SAUAL, WBLL#1, Kachu#1, BAV92//IRENA/KAUZ, FRANCOLIN#1, AMUR, ROLF07, FRET2, BABAX, and BORL14. The parental line SAUAL was the most common parent in group G–IV. The parental lines with TRCH/SRTU//KACHU cross in their genetic backgrounds dominated group G–V.

### Marker–Trait Associations of Spot Blotch

**FIGURE 4:**
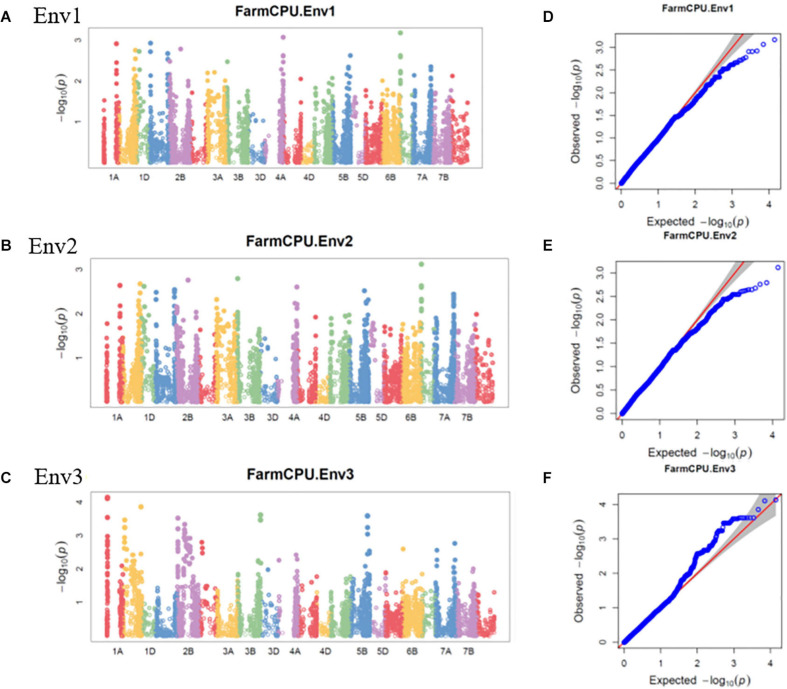
Manhattan plots representing 21 chromosomes showing the significant markers detected by FarmCPU model using BLUP values for spot blotch in **(A)** Env1, **(B)** Env2, and **(C)** Env3 environments. Quantile–quantile (Q–Q) plots for spot blotch in Env1 **(D)**, Env2 **(E)**, and Env3 **(F)** environments showing expected null distribution of *p-values*, assuming no associations, represented as a red solid line v/s; distribution of *p-values* observed using FarmCPU model represented as the blue dots.

**FIGURE 5:**
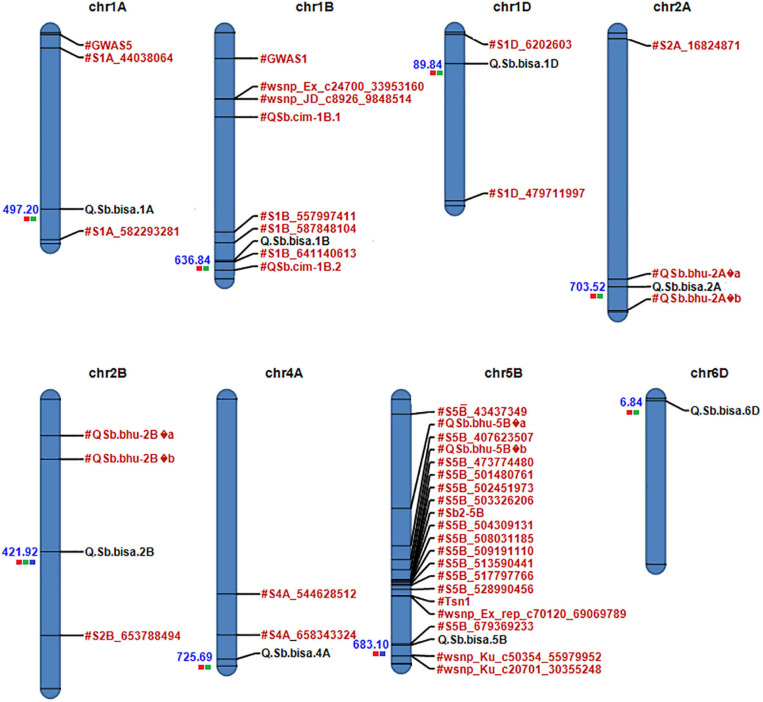
Physical map of candidate QTLs on 1A, 1B,1D, 2A, 2D, 4A, 5B, and 6D chromosomes. Significant QTLs/associated with spot blotch are highlighted in black, while previously reported QTLs/markers are labeled in red (the red color box next to QTLs for Env1, green for Env2, and blue for Env3). The physical position is based on IWGSC 2018 (RefSeq v1.0).

**FIGURE 6:**
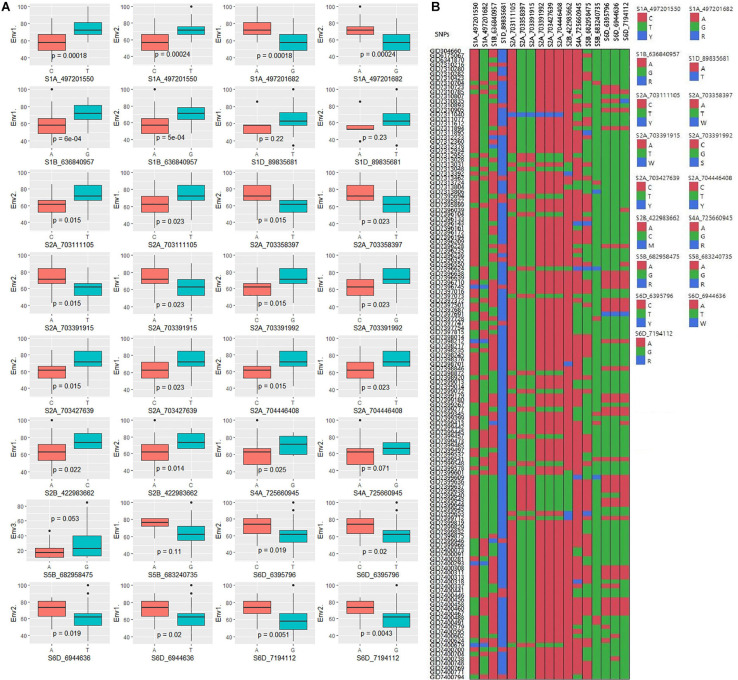
**(A)** Boxplots showing the effect of phenotypic variation between the two alleles of the SNPs for disease score of bread wheat; the Kruskal–Wallis test was used to determine the significant differences between the mean values of two alleles. **(B)** Cell plot of the distribution of the different alleles of significant SNPs in the advanced wheat breeding lines.

The GWAS of spot blotch resistance was performed based on the disease scores collected at adult plant stages. A *p-value* < 0.003 was used as a threshold to identify significant MTAs. The GWAS results from three environments are given in Figure 4. A total of 29 significant MTAs appeared in a minimum of two environments belonging to eight QTLs on chromosome 1A, 1B, 1D, 2A, 2B, 4A, 5B, and 6D (Figure 5). The allele effects of those QTLs ranged from −31.37% (Env1) to 30.67% (Env2) while the allele effects for individual environments ranged from −31.37 to 30.07%, −22.83 to 30.67%, and −23.30 to 23.24% in Env1, Env2, and Env3, respectively (Table 2). The largest allele effect is explained by the SNP S2B_422983662 located on chromosome 2B in Env2 (30.67%) (Table 2). The allele effects for alternative alleles from each of the associated SNP markers were plotted (Figure 6). We detected a significant variance in the mean values of the favorable alleles that led to an increase in resistance varying from 12.5 to 70% for spot blotch. The “Kruskal–Wallis” test was used to determine whether there are significant differences between the mean values of two alleles. Major alleles have lower mean values compared to minor alleles except for two QTLs (*Q.Sb.bisa-1D* and *Q.Sb.bisa-5B*), where minor alleles are found to be effective (Figure 6 and Table 2).

**TABLE 2:** Summary of the significant SNPs associated with spot blotch resistance.

**Names of QTLs**	**SNP**	**Alleles**	**Effective allele**	**Env**	**Position (Mb)**	***p*-value**	**MAF**	**Marker effect**	**Effect 10X**
*Q.Sb.bisa-1A* (Env 1 and 2)	S1A_497201550	C/T	C	Env1	497.20	0.001232	0.28	1.88	18.83
			C	Env2	497.20	0.002382	0.28	1.83	18.26
	S1A_497201682	G/A	G	Env1	497.20	0.001232	0.28	–1.88	–18.83
			G	Env2	497.20	0.002382	0.28	–1.83	–18.26
*Q.Sb.bisa-1B* (Env 1 and 2)	S1B_636840957	A/G	A	Env1	636.84	0.001754	0.37	1.61	16.14
			A	Env2	636.84	0.002104	0.37	1.63	16.27
*Q.Sb.bisa-1D* (Env 1 and 2)	S1D_89835681	T/A	A	Env1	89.84	0.001896	0.13	2.40	23.95
			A	Env2	89.84	0.002417	0.13	2.40	24.00
*Q.Sb.bisa-2A* (Env 1 and 2)	S2A_703111105	C/T	C	Env2	703.11	0.002881	0.16	2.28	22.83
			C	Env1	703.11	0.002946	0.16	2.22	22.23
	S2A_703358397	T/A	T	Env2	703.36	0.002881	0.16	–2.28	–22.83
			T	Env1	703.36	0.002946	0.16	–2.22	–22.23
	S2A_703391915	T/A	T	Env2	703.39	0.002881	0.16	–2.28	–22.83
			T	Env1	703.39	0.002946	0.16	–2.22	–22.23
	S2A_703391992	C/G	C	Env2	703.39	0.002881	0.16	2.28	22.83
			C	Env1	703.39	0.002946	0.16	2.22	22.23
	S2A_703427639	C/T	C	Env2	703.43	0.002881	0.16	2.28	22.83
			C	Env1	703.43	0.002946	0.16	2.22	22.23
	S2A_704446408	C/T	C	Env2	704.45	0.002881	0.16	2.28	22.83
			C	Env1	704.45	0.002946	0.16	2.22	22.23
*Q.Sb.bisa-2B* (Env1,2, and 3)	S2B_419320960	C/T	*	Env3	419.32	0.002276	0.46	2.32	23.25
	S2B_419456700	T/C	*	Env3	419.46	0.002138	0.45	–2.33	–23.30
	S2B_420723687	G/T	*	Env3	420.72	0.002497	0.46	2.30	23.03
	S2B_421708152	A/G	*	Env3	421.71	0.002655	0.46	2.30	22.98
	S2B_422031949	T/C	*	Env3	422.03	0.002655	0.46	–2.30	–22.98
	S2B_422190984	C/A	*	Env3	422.19	0.002655	0.46	–2.30	–22.98
	S2B_422191033	A/G	*	Env3	422.19	0.002655	0.46	2.30	22.98
	S2B_422507900	C/T	*	Env3	422.51	0.002655	0.46	2.30	22.98
	S2B_422983662	A/C	A	Env1	422.98	0.001657	0.11	3.01	30.07
			A	Env2	422.98	0.001748	0.11	3.07	30.68
	S2B_423094651	G/A	*	Env3	423.09	0.002655	0.46	–2.30	–22.98
	S2B_423836280	C/G	*	Env3	423.84	0.002655	0.46	2.30	22.98
*Q.Sb.bisa-4A* (Env 1 and 2)	S4A_725538462	C/T	*	Env1	725.54	0.002455	0.21	1.94	19.41
	S4A_725660945	A/G	A	Env1	725.66	0.000855	0.24	2.02	20.18
			A	Env2	725.66	0.002464	0.24	1.89	18.86
	S4A_725880148	A/G	*	Env1	725.88	0.002388	0.21	1.97	19.72
*Q.Sb.bisa-5B* (Env1 and 3)	S5B_682958475	G/A	A	Env3	682.96	0.002912	0.46	2.14	21.41
	S5B_683240735	G/A	G	Env1	683.24	0.002373	0.07	–3.14	–31.37
*Q.Sb.bisa-6D* (Env 1 and 2)	S6D_6395796	T/C	T	Env2	6.4	0.002335	0.27	–1.78	–17.77
			T	Env1	6.4	0.002360	0.27	–1.73	–17.32
	S6D_6944636	T/A	T	Env2	6.94	0.002511	0.27	–1.77	–17.66
			T	Env1	6.94	0.002589	0.27	–1.72	–17.18
	S6D_7194112	G/A	G	Env1	7.19	0.000669	0.27	–1.99	–19.94
			G	Env2	7.19	0.000763	0.27	–2.02	–20.23

Env, environment; MAF, minor allele frequency. *Asterisk sign in the effective allele column represents that the allelic effect of the two alleles was not significantly different.

### Physical Mapping

**FIGURE 7:**
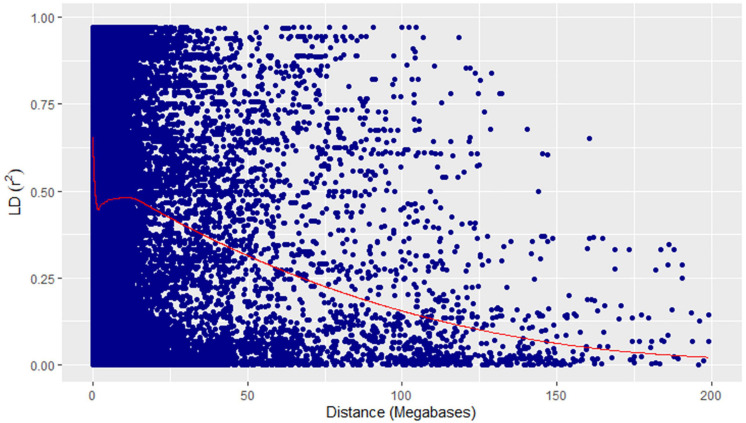
Linkage disequilibrium decay plot (pairwise) showing squared allele-frequency correlation (*r*^2^) vs the megabase pair distance (Mbp) between the pairs of GBS markers showing in blue dots. Megabase pair distances are shown on the *x*-axis and coefficient of determination (*r*^2^) on the *y*-axis. The red line represents the loess curve fitted on the decay plot.

A physical map was prepared using the most significant QTLs placed on eight hexaploid wheat chromosomes. The significant QTLs were clustered into eight linkage groups (Figure 5). A group was considered to be different based on critical LD (*r*^2^ ∼ 0.2) (Figure 7). We observed one linkage group on each of 1A, 1B, 1D, 2A, 2B, 4A, and 6D. The maximum number of significant MTAs in two environments (six MTAs) is observed on chromosome 2A (Table 2).

### Putative Candidate Genes and Annotations

The significant QTLs identified from the GWAS analysis were further studied for the known candidate genes relevant to disease resistance using the recently annotated wheat reference sequence (RefSeq V1.0). We identified NBS-LRR, MADS transcription factor, and 34 other plant protein families across chromosomal regions associated with significant QTLs in the study. The SNP S1A_497201550 associated with *Q.Sb.bisa-1A* identified in Env1 and Env2 on chromosome 1A was located between the *TraesCS1A01G303600*, a gene that encodes LURP-one-like protein, and *TraesCS1A01G303700*, the gene encoding GTP cyclohydrolase 1. Similarly, the SNP S1B_636840957 belongs to *Q.Sb.bisa-1B* identified in Env1 and Env2 on chromosome 1B with GeneID *TraesCS1B01G409800* that encodes 60S ribosomal protein L35a-like protein and *TraesCS1B01G40900* encoding transmembrane protein (Table 3). The SNP S1D_89835681, which belongs to the *Q.Sb.bisa-1D* identified in Env1 and Env2, was located close to *TraesCS1D01G101800* associated with Pre-rRNA-processing protein TSR2 and *TraesCS1D01G101900* encoding glucan 1,3 beta-glucosidase. Similarly, the annotation of *Q.Sb.bisa-2A* with SNPs S2A_703111105, S2A_703358397, S2A_703391915, S2A_703391992, and S2A_703427639 revealed that the associated genes encode the proteins related to senescence-associated family protein.

**TABLE 3:** SNPs with the corresponding proteins and possible function elucidated based on the gene annotation using wheat reference sequence (RefSeq V1.0) annotation database.

**QTLs and Env**	**SNP**	**Gene ID**	**Dist. from SNP (in bp)**	**Gene annotation**
*Q.Sb.bisa-1A* (Env 1 and 2)	S1A_497201550	TraesCS1A01G303600	+9400	LURP-one-like protein
		TraesCS1A01G303700	−23,504	GTP cyclohydrolase 1
	S1A_497201682	TraesCS1A01G303600	+9532	LURP-one-like protein
		TraesCS1A01G303700	−23,372	GTP cyclohydrolase 1
*Q.Sb.bisa-1B* (Env 1 and 2)	S1B_636840957	TraesCS1B01G409800	+2658	60Sribosomal protein L35a-like protein
		TraesCS1B01G409900	−19,389	Transmembrane protein, putative
*Q.Sb.bisa-1D* (Env 1 and 2)	S1D_89835681	TraesCS1D01G101800	+237,973	Pre-rRNA-processing protein TSR2
		TraesCS1D01G101900	−312,998	Glucan 1,3-beta-glucosidase
*Q.Sb.bisa-2A* (Env 1 and 2)	S2A_703111105	TraesCS2A01G453900	+92	Senescence-associated family protein
		TraesCS2A01G454000	−11,947	Senescence-associated family protein
	S2A_703358397	TraesCS2A01G454400	+12,352	Senescence-associated family protein
		TraesCS2A01G454500	−23,631	Senescence-associated family protein
	S2A_703391915	TraesCS2A01G454500	+9887	Senescence-associated family protein
		TraesCS2A01G454600	−10,955	Senescence-associated family protein
	S2A_703391992	TraesCS2A01G454500	+9964	Senescence-associated family protein
		TraesCS2A01G454600	−10,878	Senescence-associated family protein
	S2A_703427639	TraesCS2A01G454600	+24,769	Senescence-associated family protein
		TraesCS2A01G454700	−20,520	senescence-associated family protein
	S2A_704446408	TraesCS2A01G455500	+427,884	Cortactin-binding protein 2
		TraesCS2A01G455600	−307,046	Lectin receptor kinase
*Q.Sb.bisa-2B* (Env1,2, and 3)	S2B_419320960	TraesCS2B01G299000	+365,182	SNF1-related protein kinase regulatory subunit gamma 1
		TraesCS2B01G299100	−717	Cotton fiber-like protein
	S2B_419456700	TraesCS2B01G299100	+135,023	Cotton fiber-like protein
		TraesCS2B01G299200	−45,804	Amino acid transporter, putative
	S2B_420723687	TraesCS2B01G299700	+520,847	MYND type zinc finger protein
		TraesCS2B01G299800	−358,751	RNA binding protein
	S2B_421708152	TraesCS2B01G300100	+3383	UPF0250 protein mma_3250
		TraesCS2B01G300200	−623	Isocitrate lyase
	S2B_422031949	TraesCS2B01G300200	+323,174	Isocitrate lyase
		TraesCS2B01G300300	−202,765	Auxin response factor
	S2B_422190984	TraesCS2B01G300200	+482,209	Isocitrate lyase
		TraesCS2B01G300300	−43,730	Auxin response factor
	S2B_422191033	TraesCS2B01G300200	+482,258	Isocitrate lyase
		TraesCS2B01G300300	−43,681	Auxin response factor
	S2B_422507900	TraesCS2B01G300400	+7502	DNAprimase large subunit
		TraesCS2B01G300500	−458,849	MADS box transcription factor
	S2B_422983662	TraesCS2B01G300500	+16,913	MADS box transcription factor
		TraesCS2B01G300600	−114,123	Transmembrane protein, putative
	S2B_423094651	TraesCS2B01G300500	+127,902	MADS box transcription factor
		TraesCS2B01G300600	−3134	Transmembrane protein, putative
	S2B_423836280	TraesCS2B01G301100	+868	Chaperone protein dnaJ
		TraesCS2B01G301200	−63,852	Zinc knuckle family protein, expressed
*Q.Sb.bisa-4A* (Env 1 and 2)	S4A_725538462	TraesCS4A01G459600	+272,847	Protein phosphatase 2C
		TraesCS4A01G459700	−33,166	Seed maturation protein
	S4A_725660945	TraesCS4A01G460000	+597	Eyes absent-like protein
		TraesCS4A01G460100	−8438	Cytochrome P450
	S4A_725880148	TraesCS4A01G460800	+19,993	Invertase inhibitor
		TraesCS4A01G460900	−13,027	Invertase inhibitor
*Q.Sb.bisa-5B* (Env1 and 3)	S5B_682958475	TraesCS5B01G521000	+5865	Transmembrane protein, putative
		TraesCS5B01G521100	−150,684	Calmodulin-binding transcription activator
	S5B_683240735	TraesCS5B01G521200	+100,340	Receptor kinase 1
		TraesCS5B01G521300	−99,434	NBS-LRR disease resistance protein family-1
*Q.Sb.bisa-6D* (Env 1 and 2)	S6D_6395796	TraesCS6D01G015700	+25,746	Leucine-rich repeat receptor-like protein kinase family protein
		TraesCS6D01G015800	−29,399	Leucine-rich repeat receptor-like protein kinase family protein
	S6D_6944636	TraesCS6D01G016400	+120,493	Transposon protein, putative, CACTA, En/Spm subclass
		TraesCS6D01G016500	−19,307	Centromere O
	S6D_7194112	TraesCS6D01G017300	+30,119	Auxin transport protein BIG
		TraesCS6D01G017400	−15,620	Polygalacturonase-1 non-catalytic beta subunit

*Distance from the SNP represents distance of the start site of gene to SNP linked with QTL, where the (+) sign represents that the gene was found downstream of the SNP and the (–) sign represents that the gene was found upstream.*

Interestingly *Q.Sb.bisa-2B* was detected in all three environments. The Gene IDs of S2B_422983662 from *Q.Sb.bisa-2B* encode MADS-box gene (*TraesCS2B01G300500*), and *TraesCS2B01G300600* encodes a transmembrane protein. The *Q.Sb.bisa-4A* on chromosome 4A with SNP S4A_725660945 encodes *TraesCS4A01G460000* Eyes absent-like protein and (*TraesCS4A01G460100*) Cytochrome P450. The *Q.Sb.bisa-5B* carrying SNP S5B_682958475 and SNP S5B_683240735. Gene IDs (*TraesCS5B01G521000* and *TraesCS5B01G521100*) from SNP S5B_682958475 encode transmembrane protein and calmodulin-binding transcription. SNP S5B_683240735 mapped between *TraesCS5B01G521200* and *TraesCS5B01G521300* encodes receptor kinase 1 and NBS-LRR disease resistance protein family-1, respectively. The *Q.Sb.bisa-6D* of SNP S6D_6395796 detected in Env1 and Env2 lies between *TraesCS6D01G015700* and *TraesCS6D01G015800*. Those genes encode *Leucine-rich repeat receptor-like protein kinase* associated with *TaWRKY76* and *TaWRKY62*. The largest allele effect was explained by the QTL located on chromosome 2B in the gene region that encodes the MADS-box transcription factor and transmembrane protein. However, the QTLs at 1A, 1B, 1D, 2A, 2B, 4A, 5B, and 6D chromosomes are found to be involved directly in the disease resistance mechanism (Table 3).

## Discussion

### Phenotypic Evaluation for Spot Blotch

The field trials were conducted at BISA research farm, Pusa, in India for two consecutive crop seasons 2019 (Env1) and 2020 (Env2). The spot blotch data recorded at BARI, Jamalpur, in Bangladesh during the 2017 crop season was included in the analysis and named Env3. Both the locations fall under the traditional, warmer wheat-growing regions belonging to Mega-Environment 5, characterized by hot and humid conditions as per CIMMYT’s system for classifying wheat-growing environments in developing countries (van Ginkel and Rajaram, 1993). The average temperature during the wheat plant reproductive phase at Jamalpur and Pusa is higher than 19°C, with high relative humidity, and serves as a congenial environment for the pathogen (Supplementary Table S3). The spot blotch disease incidence was recorded as a percentage of the infected leaf area at three different growth stages to minimize the chances of disease escape. Since the susceptible parent displayed the highest disease severity at GS77 on the Zadoks scale (Zadoks et al., 1974), the scoring at this stage (GS77) was used in further data analysis. Although the testing sites are considered as hot spots for spot blotch (Sharma et al., 2007b), to avoid escaping of genotypes from the pathogen, the highly susceptible cultivar Sonalika was planted in alleys. The BLUPs of disease severity in three environments ranged from 2.65 to 39.35 among advanced wheat breeding lines. The right-tailed skewness of the data in Env3 highlights a likely impact of low disease pressure under natural infection without inoculation in Bangladesh. The nearly continuous distribution of disease scores in all environments indicates the quantitative nature of resistance being caused by the additive effect of various QTLs/genes. A similar trend was reported in some of the earlier findings, where more than two genes (Joshi et al., 2004; Kumar et al., 2007, 2009) and multiple alleles with minor effects (Neupane et al., 2007; Ayana et al., 2018; Singh et al., 2018) were found to be controlling the spot blotch disease resistance.

We observed significant genetic variation for disease susceptibility in the population. The genetic variances and high heritabilities for spot blotch were comparable with earlier findings in wheat (Sharma et al., 1997; Joshi et al., 2004). Despite significant genotype × environment interactions, we observed high broad-sense heritability for disease across environments (Table 1), highlighting the considerable genetic variation germane for further genetic dissection. The environmental interactions might ascribe to temperature differences at the time of disease development or the difference in the pathogen isolates prevalent in the North Eastern Plains Zone (NEPZ) of India and Bangladesh as well as the varied weather conditions within the location (Supplementary Table S3).

### Principal Component Analysis

Twelve groups formed in the phylogeny were genetically distinct based on PCA (Figure 3). It shows that the advanced lines used in the current study had considerable diversity to identify multiple alleles due to the higher number of resistance sources. Common parent TRCH/SRTU//KACHU are found in group G–V, while the largest group (G–X) consists of lines with mixed pedigrees dominated by TEPOKA, TRCH, SAUAL, WBLL#1, KACHU#1, BAV92//IRENA/KAUZ, FRANCOLIN#1, AMUR, ROLF07, FRET2, BABAX, and BORL14, providing the clues for the resistant genetic resources. Wheat accessions FRANCOLIN, MUCUY, TACUPETO F2001, NADI, BOKOTA, KAUZ, ROLF07, and KACHU were the major contributors to the parentage of most of the genotypes. These results confirm the notion that some elite cultivars have been frequently utilized in the pedigree of germplasm.

### SNP Effects in Different Environments of Spot Blotch

Several spot blotch resistance QTLs have been reported on different chromosomes (Neupane et al., 2007; Sharma et al., 2007a; Gonzalez-Hernandez et al., 2009; Kumar et al., 2009, 2010, 2015; Adhikari et al., 2012; Lillemo et al., 2013; Zhuang et al., 2013; Gurung et al., 2014; Lu et al., 2016). However, only four major QTLs designated as *Sb1* on 7D (Lillemo et al., 2013), *Sb2* on 5B (Kumar et al., 2015), *Sb3* on 3B (Lu et al., 2016), and *Sb4* on 4BL (Zhang et al., 2020) are well described. We also observed consistent chromosomal regions on 2B and 5B, which were detected in two or all three environments (Table 2). Regardless of phenotypic effects explained by an allele, most of the wheat chromosomes have been reported for their contribution to spot blotch disease resistance (Sharma et al., 2007a; Kumar et al., 2009, 2010; Adhikari et al., 2012; Lillemo et al., 2013; Gurung et al., 2014; Zhu et al., 2014; Lu et al., 2016; Gupta et al., 2017; Ayana et al., 2018; Singh et al., 2018; Zhang et al., 2020). The broad range of environmental conditions at our field-testing sites allowed us to capture considerable genetic variation underlying spot blotch resistance. After GWAS analysis, significant SNP markers on eight chromosomes harboring QTL regions forming eight QTLs were physically mapped and used for further analysis. The previously reported genes and markers linked to spot blotch resistance were physically mapped along with the most significant SNPs detected in this study to identify if any new regions were uncovered (Figure 5).

To study the importance of significant SNPs in disease resistance, we annotated all SNPs using the wheat reference genome annotation (IWGSC Ref Seq v1.0). The literature was mined intensively to look for the putative functions of those genes/proteins. We found that the functions of several genes were strongly associated with disease resistance across the year and environments (Table 3). Two significant chromosomal regions/QTLs on 2B and 5B were consistent between Pusa, India, and Jamalpur, Bangladesh. This may be due to the prevalence of the most aggressive isolate of spot blotch pathogen (isolate no. ICMP 13584, Auckland, New Zealand) common in South Asia (Chaurasia et al., 2000). Although we were able to capture large allelic effects ranging from −31.4% (Env1-5B) to 30.7% (Env2-2B) for spot blotch disease severity, some genomic regions of small effect may remain undetected due to the multiple testing criteria of GWAS.

In our study, eight diverse genomic regions were found to be associated with chromosomes 1A, 1B, 1D, 2A, 2B, 4A, 5B, and 6D. The present study not only reports the region on 1A elucidating up to 18.8% of phenotypic variation but also provides insight on the markers S1A_497201550 and S1A_497201682 by gene annotation. The previous studies (Adhikari et al., 2012; Jamil et al., 2018; Bainsla et al., 2020) on spot blotch reported the chromosomal region on 1A explained 2.1–10% of phenotypic variation. Both SNP markers (S1A_497201550 and S1A_497201682) were associated with LURP-one-like protein that mediates resistance by coordinated transcriptional upregulation of plant defense genes (Knoth et al., 2009). It is noteworthy that the SNP marker S1B_636840957 on chromosome 1B was associated with spot blotch severity, where the annotation study revealed that S1B_636840957 is close to transmembrane protein, which regulates fungal development and pathogenicity via the MAPK module (Gu et al., 2015). Another significant marker S1D_8983568 accounted for 24.0% of the allelic effect, consistent with the earlier studies (Bainsla et al., 2020). However, the QTL reported in our study and Bainsla et al. (2020) was more than 83 Mb apart at chromosome 1D.

The *Q.Sb.bisa-2A* at chromosome 2A between the region (703.11–704.44 Mb) was found to be linked with the senescence-associated family protein. For instance, if one allele is involved with senescence, then the alternate allele is involved with the “stay-green” trait in wheat. Stay-green has been reported to be associated with spot blotch disease resistance (Joshi et al., 2007a; Rosyara et al., 2008). The present results and other independent studies also indicated the importance of this chromosome region in spot blotch disease resistance (Joshi et al., 2007a; Rosyara et al., 2008; Bainsla et al., 2020).

Kumar et al. (2010) analyzed two bi-parental mapping populations and reported four QTLs for SB resistance including chromosomes 2AS explaining up to 22.7% of phenotypic variation. The other interesting *Q.Sb.bisa-2B* lie between MADS-box genes that were reported to be differentially expressed in response to stripe rust pathogen in wheat (Guo et al., 2013) and transmembrane protein, which regulates fungal development and pathogenicity via the MAPK module (Gu et al., 2015). Similar to 1D, the *Q.Sb.bisa-2B* on 2B mapped nearly 231 Mb apart from the QTL reported by Kumar et al. (2010) and Bainsla et al. (2020). The SNP S4A_725660945 of *Q.Sb.bisa-4A* is associated with wheat Cytochrome P450 family protein. This protein enhances resistance to mycotoxin, namely, deoxynivalenol (DON) and grain yield (Gunupuru et al., 2018). This QTL is in agreement with the earlier finding of Ayana et al. (2018) which was mapped in the same chromosomal region but physically a few Mb away. Since QTL mapping is based on recombination frequency, the possibility of both the QTLs/SNPs being in the same chromosomal region may not be ruled out.

The markers of *Q.Sb.bisa-5B* QTL (S5B_682958475 and S5B_683240735) were associated with calmodulin-binding transcription activator, receptor kinase 1, and NBS-LRR disease resistance protein family-1 which are involved in the defense response of wheat to *Puccinia triticina* (Wang Y. et al., 2019) and fungal pathogen *Zymoseptoria tritici*. On the other hand, the NBS-LRR disease resistance protein family is well known to contribute to fungal disease resistance (He et al., 2018). The QTL on 5B, named the *Sb2* gene, has been studied in detail (Lu et al., 2016), known to interact with the *Tsn1* gene, conferring susceptible reaction to tan spot and *Septoria nodorum* blotch (McDonald et al., 2018). The gene *ToxA* virulent to *Tsn1* exists in both *Pyrenophora tritici-repentis* and *Parastagonospora nodorum* which confer susceptible reaction to tan spot and *S. nodorum* blotch, respectively (Singh et al., 2018). Friesen et al. (2018) demonstrated the major effects of the *Tsn1* locus on chromosome 5B. However, the importance of *Tsn1* in spot blotch disease resistance under field conditions is not known. Another QTL for spot blotch resistance was mapped earlier in the same region by Jamil et al. (2018).

**FIGURE 8:**
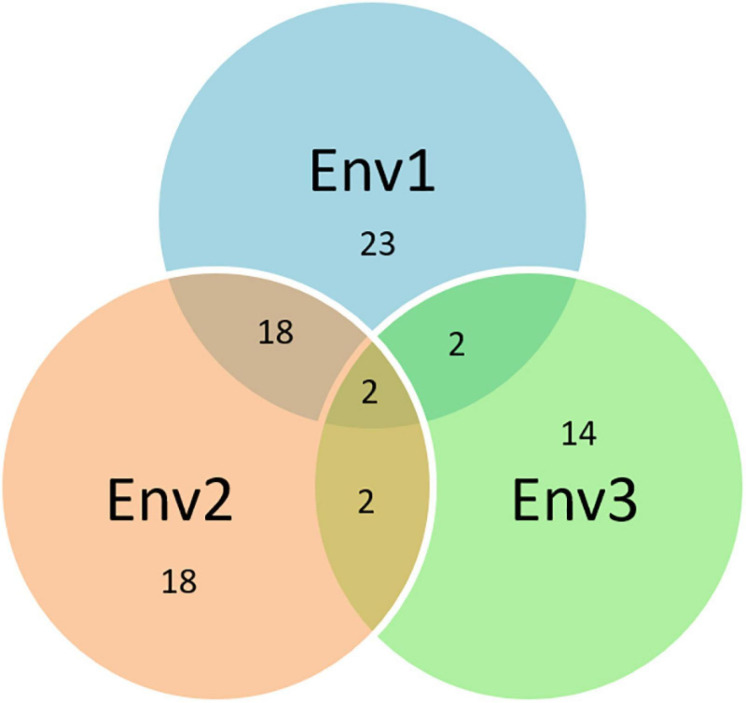
Venn diagram based on the common protein synthesized by the same genes associated with different SNPs over years (the numbers in the circle represent the total numbers of proteins in the respective environments and the numbers at intersects represent the common proteins between the environments).

The *Q.Sb.bisa-6D* carrying SNP S6D_6395796 on chromosome 6D had an allelic effect of −17.8% with the stable response in two environments and annotated to be located close to a gene that synthesizes Leucine-rich repeat receptor-like protein kinase family protein, which plays an important role in disease resistance. The LRR-Like kinase gene associated with *TaWRKY76* and *TaWRKY62* plays a positive role in wheat high-temperature plant resistance to *Puccinia striiformis* f. sp. *tritici* (Wang J. et al., 2019). The 6D chromosome has earlier been identified to impart resistance against spot blotch in QTL mapping but via different genomic regions, i.e., near the centromeric region (Kumar et al., 2009, 2010) and proximal region of 6DS (Singh et al., 2018). The exact physical regions of these QTLs could not be estimated as they were either based on SSR markers or a genetic map. The consistency of SNPs on eight chromosomes (1A, 1B, 1D, 2A, 2B, 4D, 5B, and 6D) in a minimum of two or all environments indicates their potential significance in the breeding of disease-resistant varieties. Further, the resistance mechanism through protein annotation was confirmed where the same or common gene/protein family was identified independently in different environments (Figure 8). With additional validation, these genetic regions reported in this study can potentially be used in fine mapping and map-based cloning to further characterize the mechanisms of spot blotch disease resistance.

## Conclusion

We identified genetic regions underlying spot blotch resistance in the elite spring wheat genotypes. The variable conditions at three field environments in India and Bangladesh allowed us to capture the considerable phenotypic variation for spot blotch disease with the GWAS resulting in a total of eight QTLs belonging to eight wheat chromosomes. The literature mining of the functional gene annotations of identified SNPs encoding the single protein or protein family directly or indirectly involved in disease resistance has led to the identification of putative target genes and functions to identify the disease-resistance mechanism. The new QTLs appeared on chromosomes 1A (497.2), 1D (89.84), 2B (421.92), and 6D (6.84) associated with many disease resistance family proteins. The SNP on chromosome 2A was found to be associated with a known gene that encodes “senescence-associated family protein” and is directly involved in spot blotch resistance. The results are of importance for the breeders in developing spot blotch-resistant varieties targeting the South Asian wheat-growing regions. Given the aggressive pathogen spread and food security concerns, the breeding programs in South Asia could benefit from the present study. The mapping of favorable alleles can facilitate introgression of the alleles into present-day elite cultivars to impart disease resistance. It is apparent from the results that some individual alleles cumulatively contributed as high as 70% for spot blotch disease resistance. Additional investigations are underway, which would further confirm the importance of these chromosomal regions/genes associated with spot blotch.

## Data Availability Statement

The datasets presented in this study can be found in Supplementary Material.

## Author Contributions

VT wrote the initial draft of the manuscript. VT, DS, and GD analyzed the data. RS provided the breeding material. JP provided genotyping data. VT and MR managed field trials and recorded phenotypic data. PB, SK, and PS provided scientific inputs during the initial manuscript drafting. VT, DS, UK, GD, and BT revised the manuscript. UK designed the experiment. UK, AJ, and BT supervised the research project. All authors read and approved the final manuscript.

## Conflict of Interest

The authors declare that the research was conducted in the absence of any commercial or financial relationships that could be construed as a potential conflict of interest.

## Supplementary Material

Supplementary Table 1 List of 139 genotypes with clustering information used in the present study.

Supplementary Table 2 Statistical analysis of spot blotch incidence in 139 wheat genotypes evaluated at two locations in three environments.

Supplementary Table 3 Monthly average, minimum, and maximum temperatures at BISA, Pusa in India.
