# Supplementary material for: New QTLs for Spot Blotch Disease Resistance in Wheat (Triticum aestivum L.) Using Genome-Wide Association Mapping
Source: Front Genet. 2021 Jan 14;11:613217. doi: 10.3389/fgene.2020.613217 (PMC7841440; doi:10.3389/fgene.2020.613217)
Supplement: Supplementary Table 1 — List of 139 genotypes with clustering information used in the present study. [file Table_1.DOCX]

**Table S1**. List of 139 Genotypes with clustering information used in present study

| **GID** | **Cluster** | **Pedigree** |
| --- | --- | --- |
| GID304660 | 1 | HD2733 |
| GID6175067 | 5 | NADI |
| GID6341870 | 7 | MUCUY |
| GID7310216 | 9 | BECARD/AKURI/4/WBLL1*2/BRAMBLING//JUCHI/3/WBLL1*2/BRAMBLING |
| GID7310280 | 3 | BORL14//KFA/2*KACHU |
| GID7310282 | 4 | BORL14//KFA/2*KACHU |
| GID7310423 | 6 | SUP152/BAJ #1/3/KACHU #1/KIRITATI//KACHU |
| GID7310704 | 9 | MUU/KBIRD/3/PRL/2*PASTOR*2//FH6-1-7 |
| GID7310723 | 1 | BECARD/QUAIU #1//BORL14 |
| GID7310785 | 9 | BECARD//ND643/2*WBLL1/4/KIRITATI//ATTILA*2/PASTOR/3/AKURI |
| GID7310807 | 9 | SITE/MO//PASTOR/3/TILHI/4/MUNAL #1/5/MUNAL/6/MUCUY |
| GID7310835 | 11 | ND643//2*ATTILA*2/PASTOR/3/WBLL1*2/KURUKU/4/WBLL1*2/BRAMBLING/6/BABAX/LR42//BABAX*2/3/KUKUNA/4/CROSBILL #1/5/BECARD |
| GID7310893 | 9 | FRET2/TUKURU//FRET2/3/MUNAL #1/4/SUP152/AKURI//SUP152 |
| GID7310905 | 4 | KACHU/KIRITATI//BORL14 |
| GID7311040 | 10 | TEPOCA+LR34/ATTILA//TILHI/3/ATTILA*2/PBW65/4/ATTILA*2/PBW65*2//KACHU/5/AMUR |
| GID7311077 | 3 | ALTAR 84/AE.SQ//OPATA/3/2*WH 542/7/VEE#8//JUP/BJY/3/F3.71/TRM/4/BCN/5/KAUZ/6/MILAN/KAUZ/8/ATTILA*2/PBW65/9/BAV92//IRENA/KAUZ/3/HUITES*2/4/CROC_1/AE.SQUARROSA (224)//KULIN/3/WESTONIA/10/WBLL1*2/BRAMBLING//KACHU |
| GID7311612 | 12 | MUNAL #1/FRANCOLIN #1/4/KZA//WH 542/2*PASTOR/3/BACEU #1/5/MUNAL*2//WAXWING*2/TUKURU |
| GID7311894 | 1 | FRANCOLIN #1/YANAC*2/3/PBW343*2/KUKUNA*2//FRTL/PIFED |
| GID7311895 | 7 | FRANCOLIN #1/YANAC*2/3/KINGBIRD #1//INQALAB 91*2/TUKURU |
| GID7312223 | 2 | YAV_3/SCO//JO69/CRA/3/YAV79/4/AE.SQUARROSA (498)/5/LINE 1073/6/KAUZ*2/4/CAR//KAL/BB/3/NAC/5/KAUZ/7/KRONSTAD F2004/8/KAUZ/PASTOR//PBW343/9/PBW343*2/KUKUNA*2//FRTL/PIFED/10/KIRITATI//PRL/2*PASTOR/5/OASIS/SKAUZ//4*BCN/3/PASTOR/4/KAUZ*2/YACO//KAUZ/6/KIRI |
| GID7312360 | 3 | AMUR/3/KINGBIRD #1//INQALAB 91*2/TUKURU |
| GID7312378 | 10 | TRCH/5/REH/HARE//2*BCN/3/CROC_1/AE.SQUARROSA (213)//PGO/4/HUITES/6/SAUAL/3/C80.1/3*BATAVIA//2*WBLL1/4/SITE/MO//PASTOR/3/TILHI |
| GID7312934 | 12 | WBLL1/KUKUNA//TACUPETO F2001/3/BAJ #1*2/4/AMUR |
| GID7312955 | 7 | WBLL1*2/4/YACO/PBW65/3/KAUZ*2/TRAP//KAUZ/5/KACHU #1/6/PBW343*2/KUKUNA*2//FRTL/PIFED/7/PBW343*2/KUKUNA*2//FRTL/PIFED |
| GID7313028 | 5 | KACHU/SAUAL*2//COPIO |
| GID7313031 | 6 | KACHU/SAUAL*2//COPIO |
| GID7313048 | 3 | SAUAL/MUTUS/3/KINGBIRD #1//INQALAB 91*2/TUKURU/4/BAJ #1/TECUE #1 |
| GID7313392 | 4 | SAUAL/4/CROC_1/AE.SQUARROSA (205)//KAUZ/3/ATTILA/5/SAUAL/8/TACUPETO F2001/6/CNDO/R143//ENTE/MEXI_2/3/AEGILOPS SQUARROSA (TAUS)/4/WEAVER/5/PASTOR/7/ROLF07/9/SAUAL/YANAC//SAUAL |
| GID7313462 | 2 | BECARD/AKURI*2/3/PBW343*2/KUKUNA*2//FRTL/PIFED |
| GID7313724 | 4 | SAUAL/YANAC//SAUAL*2/3/TACUPETO F2001/BRAMBLING*2//KACHU |
| GID7313804 | 4 | TACUPETO F2001/6/CNDO/R143//ENTE/MEXI_2/3/AEGILOPS SQUARROSA (TAUS)/4/WEAVER/5/PASTOR/7/ROLF07*2/8/SAUAL/YANAC//SAUAL |
| GID7313806 | 4 | TACUPETO F2001/6/CNDO/R143//ENTE/MEXI_2/3/AEGILOPS SQUARROSA (TAUS)/4/WEAVER/5/PASTOR/7/ROLF07*2/8/SAUAL/YANAC//SAUAL |
| GID7395694 | 6 | KACHU/SUP152 |
| GID7395822 | 10 | SAUAL/MUTUS/4/KACHU #1//WBLL1*2/KUKUNA/3/BRBT1*2/KIRITATI |
| GID7395899 | 3 | PBW343*2/KUKUNA*2//FRTL/PIFED/5/KACHU #1/3/C80.1/3*BATAVIA//2*WBLL1/4/KACHU |
| GID7396039 | 3 | KASUKO |
| GID7396104 | 5 | SITE/MO//PASTOR/3/TILHI/4/WAXWING/KIRITATI/5/KACHU #1/KIRITATI//KACHU |
| GID7396133 | 3 | BORL14//KFA/2*KACHU |
| GID7396143 | 10 | BORL14//KFA/2*KACHU |
| GID7396161 | 3 | SAUAL/YANAC//SAUAL/3/BECARD/QUAIU #1 |
| GID7396172 | 4 | PBW343*2/KUKUNA*2//FRTL/PIFED/3/KFA/2*KACHU |
| GID7396194 | 3 | TILILA/TUKURU/4/SERI.1B*2/3/KAUZ*2/BOW//KAUZ/5/KFA/2*KACHU |
| GID7396209 | 6 | WAXWING/2*ROLF07//BORL14 |
| GID7396228 | 2 | WBLL1/FRET2//PASTOR*2/3/MURGA/5/ND643//2*ATTILA*2/PASTOR/3/WBLL1*2/KURUKU/4/WBLL1*2/BRAMBLING |
| GID7396235 | 1 | KUTZ//KFA/2*KACHU |
| GID7396236 | 1 | KUTZ//KFA/2*KACHU |
| GID7396355 | 10 | WBLL1*2/SHAMA//BAJ #1/3/BORL14 |
| GID7396550 | 7 | MUCUY//MUTUS*2/TECUE #1 |
| GID7396624 | 8 | BECARD/FRNCLN/3/KACHU #1/KIRITATI//KACHU |
| GID7396638 | 12 | CHEWINK #1/CHYAK/5/UP2338*2/VIVITSI/3/FRET2/TUKURU//FRET2/4/MISR 1 |
| GID7396639 | 12 | CHEWINK #1/CHYAK/5/UP2338*2/VIVITSI/3/FRET2/TUKURU//FRET2/4/MISR 1 |
| GID7396710 | 3 | SHORTENED SR26 TRANSLOCATION/4/ATTILA/3*BCN//BAV92/3/PASTOR/5/MUNAL/6/MUTUS*2/TECUE #1 |
| GID7396745 | 4 | ATTILA/3*BCN//BAV92/3/PASTOR/4/TACUPETO F2001*2/BRAMBLING/5/PAURAQ/6/KFA/2*KACHU |
| GID7397016 | 10 | SAUAL/MUTUS/3/ATTILA*2/PBW65*2//KACHU/4/SUP152/AKURI//SUP152 |
| GID7397073 | 3 | AMUR/3/KINGBIRD #1//INQALAB 91*2/TUKURU/4/AMUR |
| GID7397372 | 2 | CNO79//PF70354/MUS/3/PASTOR/4/BAV92*2/5/HAR311/6/PBW343*2/KUKUNA*2//FRTL/PIFED/7/CNO79//PF70354/MUS/3/PASTOR/4/BAV92*2/5/HAR311 |
| GID7397501 | 3 | ATTILA*2/PBW65*2//MURGA/4/MUU #1//PBW343*2/KUKUNA/3/MUU/5/ATTILA*2/PBW65//MURGA |
| GID7397687 | 12 | WAXWING/KIRITATI*2/3/C80.1/3*BATAVIA//2*WBLL1/4/COPIO/5/ND643//2*ATTILA*2/PASTOR/3/WBLL1*2/KURUKU/4/WBLL1*2/BRAMBLING |
| GID7397691 | 12 | WAXWING/KIRITATI*2/3/C80.1/3*BATAVIA//2*WBLL1/4/COPIO/5/ND643//2*ATTILA*2/PASTOR/3/WBLL1*2/KURUKU/4/WBLL1*2/BRAMBLING |
| GID7397728 | 11 | MUNAL #1/FRANCOLIN #1/5/KIRITATI/4/2*BAV92//IRENA/KAUZ/3/HUITES/6/BECARD/FRNCLN |
| GID7397747 | 9 | FRNCLN/ROLF07/3/KACHU #1/KIRITATI//KACHU/4/FRANCOLIN #1/YANAC |
| GID7397754 | 9 | FRNCLN/ROLF07//COPIO/3/FRNCLN*2/TECUE #1 |
| GID7397815 | 11 | FRANCOLIN #1//WBLL1*2/BRAMBLING*2/3/COPIO |
| GID7398014 | 7 | BECARD/AKURI/3/KINGBIRD #1//INQALAB 91*2/TUKURU/4/BECARD/AKURI |
| GID7398212 | 8 | NADI/3/PBW343*2/KUKUNA*2//FRTL/PIFED/4/NADI |
| GID7398217 | 5 | NADI/3/KINGBIRD #1//INQALAB 91*2/TUKURU/4/NADI |
| GID7398235 | 5 | NADI/COPIO//NADI |
| GID7398245 | 5 | NADI/COPIO//NADI |
| GID7398376 | 1 | WBLL1*2/KURUKU//HEILO/3/WBLL1*2/KURUKU/4/TACUPETO F2001/BRAMBLING*2//KACHU/5/WBLL1*2/KURUKU//HEILO/3/WBLL1*2/KURUKU |
| GID7398701 | 7 | PSN/BOW//SERI/3/MILAN/4/ATTILA/5/KAUZ*2/CHEN//BCN/3/MILAN/6/WBLL1*2/SHAMA/7/SAUAL/YANAC//SAUAL |
| GID7398846 | 12 | ATTILA*2/PBW65*2//MURGA/3/FRANCOLIN #1//WBLL1*2/KIRITATI |
| GID7398872 | 5 | KACHU #1/YUNMAI 47//KACHU/5/SAUAL/3/C80.1/3*BATAVIA//2*WBLL1/4/SITE/MO//PASTOR/3/TILHI |
| GID7398920 | 8 | BOKOTA/3/KINGBIRD #1//INQALAB 91*2/TUKURU |
| GID7399013 | 9 | FRANCOLIN #1/YANAC/5/KIRITATI/4/2*BAV92//IRENA/KAUZ/3/HUITES |
| GID7399014 | 10 | FRANCOLIN #1/YANAC/5/KIRITATI/4/2*BAV92//IRENA/KAUZ/3/HUITES |
| GID7399072 | 6 | HUIRIVIS #1*2/MURGA/3/TACUPETO F2001/BRAMBLING*2//KACHU |
| GID7399179 | 12 | SEHER 06/3/PBW343*2/KUKUNA//TECUE #1 |
| GID7399180 | 12 | SEHER 06/3/PBW343*2/KUKUNA//TECUE #1 |
| GID7399267 | 12 | WHEAR//2*PRL/2*PASTOR/3/WAXBI/4/COPIO |
| GID7399277 | 7 | WHEAR//2*PRL/2*PASTOR/5/UP2338*2/SHAMA/3/MILAN/KAUZ//CHIL/CHUM18/4/UP2338*2/SHAMA/6/UP2338*2/KKTS*2//YANAC |
| GID7399345 | 1 | OTUS//WBLL1*2/TUKURU/3/2*PBW343*2/KUKUNA*2//FRTL/PIFED |
| GID7399399 | 10 | TACUPETO F2001/BRAMBLING//PVN/3/KINGBIRD #1//INQALAB 91*2/TUKURU/5/KIRITATI/4/2*BAV92//IRENA/KAUZ/3/HUITES |
| GID7399413 | 2 | TACUPETO F2001/BRAMBLING//KIRITATI/5/C80.1/3*BATAVIA//2*WBLL1/3/ATTILA/3*BCN*2//BAV92/4/WBLL1*2/KURUKU/6/ROLF07/YANAC//TACUPETO F2001/BRAMBLING |
| GID7399442 | 4 | ATTILA*2/PBW65/5/CNO79//PF70354/MUS/3/PASTOR/4/BAV92/8/2*TACUPETO F2001/6/CNDO/R143//ENTE/MEXI_2/3/AEGILOPS SQUARROSA (TAUS)/4/WEAVER/5/PASTOR/7/ROLF07 |
| GID7399445 | 12 | FRET2*2/KUKUNA//PRINIA/PASTOR/3/2*COPIO |
| GID7399457 | 10 | WBLL1*2/4/YACO/PBW65/3/KAUZ*2/TRAP//KAUZ/5/SAUAL/6/2*KINGBIRD #1//INQALAB 91*2/TUKURU |
| GID7399473 | 6 | WBLL1*2/KKTS//PASTOR/KUKUNA/3/KINGBIRD #1//INQALAB 91*2/TUKURU/5/KAUZ//ALTAR 84/AOS/3/MILAN/KAUZ/4/SAUAL |
| GID7399485 | 6 | WBLL1*2/KKTS//PASTOR/KUKUNA/3/KINGBIRD #1//INQALAB 91*2/TUKURU/5/KAUZ//ALTAR 84/AOS/3/MILAN/KAUZ/4/SAUAL |
| GID7399497 | 6 | KACHU/SAUAL*2/3/KINGBIRD #1//INQALAB 91*2/TUKURU |
| GID7399537 | 10 | AMUR*2/3/TRCH/SRTU//KACHU |
| GID7399541 | 1 | ROLF07/YANAC//TACUPETO F2001/BRAMBLING*2/5/UP2338*2/SHAMA/3/MILAN/KAUZ//CHIL/CHUM18/4/UP2338*2/SHAMA |
| GID7399548 | 10 | ROLF07*2/KIRITATI/3/2*KINGBIRD #1//INQALAB 91*2/TUKURU |
| GID7399578 | 10 | FRET2*2/SHAMA//PARUS/3/FRET2*2/KUKUNA*2/4/KINGBIRD #1//INQALAB 91*2/TUKURU |
| GID7399601 | 10 | FRET2/KUKUNA//FRET2/3/YANAC/4/FRET2/KIRITATI/5/2*BOKOTA |
| GID7399609 | 2 | FRET2/KUKUNA//FRET2/3/YANAC/4/FRET2/KIRITATI/5/2*TACUPETO F2001/BRAMBLING*2//KACHU |
| GID7399636 | 5 | TRCH/SRTU//KACHU*2/5/UP2338*2/SHAMA/3/MILAN/KAUZ//CHIL/CHUM18/4/UP2338*2/SHAMA |
| GID7399637 | 5 | TRCH/SRTU//KACHU*2/5/UP2338*2/SHAMA/3/MILAN/KAUZ//CHIL/CHUM18/4/UP2338*2/SHAMA |
| GID7399638 | 5 | TRCH/SRTU//KACHU*2/5/UP2338*2/SHAMA/3/MILAN/KAUZ//CHIL/CHUM18/4/UP2338*2/SHAMA |
| GID7399640 | 5 | TRCH/SRTU//KACHU*2/5/UP2338*2/SHAMA/3/MILAN/KAUZ//CHIL/CHUM18/4/UP2338*2/SHAMA |
| GID7399643 | 5 | TRCH/SRTU//KACHU*2/5/UP2338*2/SHAMA/3/MILAN/KAUZ//CHIL/CHUM18/4/UP2338*2/SHAMA |
| GID7399644 | 5 | TRCH/SRTU//KACHU*2/5/UP2338*2/SHAMA/3/MILAN/KAUZ//CHIL/CHUM18/4/UP2338*2/SHAMA |
| GID7399645 | 5 | TRCH/SRTU//KACHU*2/3/KINGBIRD #1//INQALAB 91*2/TUKURU |
| GID7399653 | 10 | TRCH/SRTU//KACHU*2/3/MUU #1/SAUAL//MUU |
| GID7399712 | 5 | PBW343*2/KUKUNA//SRTU/3/PBW343*2/KHVAKI/4/VORB/FISCAL//AKURI #1/5/PBW343*2/KUKUNA//SRTU/3/PBW343*2/KHVAKI |
| GID7399819 | 2 | WBLL1/3/STAR//KAUZ/STAR/4/BAV92/RAYON/5/TRAP#1/BOW/3/VEE/PJN//2*TUI/4/BAV92/RAYON*2/8/TACUPETO F2001/6/CNDO/R143//ENTE/MEXI_2/3/AEGILOPS SQUARROSA (TAUS)/4/WEAVER/5/PASTOR/7/ROLF07 |
| GID7399829 | 6 | UP2338*2/SHAMA/3/MILAN/KAUZ//CHIL/CHUM18/4/UP2338*2/SHAMA*2/5/PBW343*2/KUKUNA*2//FRTL/PIFED |
| GID7399832 | 6 | UP2338*2/SHAMA/3/MILAN/KAUZ//CHIL/CHUM18/4/UP2338*2/SHAMA*2/5/PBW343*2/KUKUNA*2//FRTL/PIFED |
| GID7399875 | 10 | BAV92//IRENA/KAUZ/3/HUITES*2/4/CROC_1/AE.SQUARROSA (224)//KULIN/3/WESTONIA/8/TACUPETO F2001/6/CNDO/R143//ENTE/MEXI_2/3/AEGILOPS SQUARROSA (TAUS)/4/WEAVER/5/PASTOR/7/ROLF07/9/BAV92//IRENA/KAUZ/3/HUITES*2/4/CROC_1/AE.SQUARROSA (224)//KULIN/3/WESTONIA |
| GID7399946 | 2 | BECARD/AKURI*2/3/PBW343*2/KUKUNA*2//FRTL/PIFED |
| GID7399966 | 3 | BECARD/AKURI*2/3/KINGBIRD #1//INQALAB 91*2/TUKURU |
| GID7400073 | 6 | SAUAL*2/6/CNDO/R143//ENTE/MEXI_2/3/AEGILOPS SQUARROSA (TAUS)/4/WEAVER/5/2*PASTOR/7/HUW234+LR34/PRINIA*2//KIRITATI/8/SAUAL/KRONSTAD F2004 |
| GID7400091 | 4 | SAUAL/3/SW89.3064//CMH82.17/SERI/4/SAUAL/5/PBW343*2/KUKUNA*2//FRTL/PIFED/6/SAUAL/KRONSTAD F2004 |
| GID7400281 | 12 | COPIO/7/SAUAL*2/6/CNDO/R143//ENTE/MEXI_2/3/AEGILOPS SQUARROSA (TAUS)/4/WEAVER/5/2*PASTOR/8/COPIO |
| GID7400293 | 12 | COPIO/7/SAUAL*2/6/CNDO/R143//ENTE/MEXI_2/3/AEGILOPS SQUARROSA (TAUS)/4/WEAVER/5/2*PASTOR/8/COPIO |
| GID7400308 | 4 | SAUAL/YANAC//SAUAL/5/UP2338*2/SHAMA/3/MILAN/KAUZ//CHIL/CHUM18/4/UP2338*2/SHAMA/6/UP2338*2/SHAMA/3/MILAN/KAUZ//CHIL/CHUM18/4/UP2338*2/SHAMA |
| GID7400311 | 8 | SAUAL/YANAC//SAUAL/5/UP2338*2/SHAMA/3/MILAN/KAUZ//CHIL/CHUM18/4/UP2338*2/SHAMA/6/UP2338*2/SHAMA/3/MILAN/KAUZ//CHIL/CHUM18/4/UP2338*2/SHAMA |
| GID7400313 | 4 | SAUAL/YANAC//SAUAL/5/UP2338*2/SHAMA/3/MILAN/KAUZ//CHIL/CHUM18/4/UP2338*2/SHAMA/6/UP2338*2/SHAMA/3/MILAN/KAUZ//CHIL/CHUM18/4/UP2338*2/SHAMA |
| GID7400318 | 8 | SAUAL/YANAC//SAUAL/3/SUP152/MUU/4/SAUAL/YANAC//SAUAL |
| GID7400337 | 11 | WBLL1*2/4/BABAX/LR42//BABAX/3/BABAX/LR42//BABAX*2/5/QUELEA |
| GID7400441 | 2 | WBLL1*2/BRAMBLING/4/BABAX/LR42//BABAX*2/3/SHAMA*2/5/PBW343*2/KUKUNA*2//FRTL/PIFED |
| GID7400446 | 1 | WBLL1*2/BRAMBLING/4/BABAX/LR42//BABAX*2/3/SHAMA*2/5/PBW343*2/KUKUNA*2//FRTL/PIFED |
| GID7400456 | 8 | THELIN/2*WBLL1/5/KAUZ//ALTAR 84/AOS/3/KAUZ/4/SW94.15464/6/2*UP2338*2/SHAMA/3/MILAN/KAUZ//CHIL/CHUM18/4/UP2338*2/SHAMA |
| GID7400458 | 8 | THELIN/2*WBLL1/5/KAUZ//ALTAR 84/AOS/3/KAUZ/4/SW94.15464/6/2*UP2338*2/SHAMA/3/MILAN/KAUZ//CHIL/CHUM18/4/UP2338*2/SHAMA |
| GID7400460 | 8 | THELIN/2*WBLL1/5/KAUZ//ALTAR 84/AOS/3/KAUZ/4/SW94.15464/6/2*UP2338*2/SHAMA/3/MILAN/KAUZ//CHIL/CHUM18/4/UP2338*2/SHAMA |
| GID7400467 | 8 | THELIN/2*WBLL1/5/KAUZ//ALTAR 84/AOS/3/KAUZ/4/SW94.15464/6/2*UP2338*2/SHAMA/3/MILAN/KAUZ//CHIL/CHUM18/4/UP2338*2/SHAMA |
| GID7400488 | 8 | KFA/5/REH/HARE//2*BCN/3/CROC_1/AE.SQUARROSA (213)//PGO/4/HUITES/6/REH/HARE//2*BCN/3/CROC_1/AE.SQUARROSA (213)//PGO/4/HUITES/7/BOKOTA/8/BOKOTA |
| GID7400491 | 8 | KFA/5/REH/HARE//2*BCN/3/CROC_1/AE.SQUARROSA (213)//PGO/4/HUITES/6/REH/HARE//2*BCN/3/CROC_1/AE.SQUARROSA (213)//PGO/4/HUITES/7/BOKOTA/8/BOKOTA |
| GID7400579 | 10 | BABAX/LR42//BABAX/3/ER2000/4/NIGHAR |
| GID7400595 | 12 | BABAX/LR42//BABAX/3/ER2000/5/GK ARON/AG SECO 7846//2180/4/2*MILAN/KAUZ//PRINIA/3/BAV92 |
| GID7400602 | 9 | TC870344/GUI//TEMPORALERA M 87/AGR/3/2*WBLL1/8/BOW/VEE/5/ND/VG9144//KAL/BB/3/YACO/4/CHIL/6/CASKOR/3/CROC_1/AE.SQUARROSA (224)//OPATA/7/PASTOR//MILAN/KAUZ/3/BAV92 |
| GID7400624 | 7 | SOKOLL/3/PASTOR//HXL7573/2*BAU/4/PANDION//FILIN/2*PASTOR/3/BERKUT |
| GID7400679 | 9 | W15.92/4/PASTOR//HXL7573/2*BAU/3/WBLL1/7/CNO79//PF70354/MUS/3/PASTOR/4/BAV92/5/FRET2/KUKUNA//FRET2/6/MILAN/KAUZ//PRINIA/3/BAV92 |
| GID7400700 | 9 | MEX94.27.1.20/3/SOKOLL//ATTILA/3*BCN/5/GK ARON/AG SECO 7846//2180/4/2*MILAN/KAUZ//PRINIA/3/BAV92 |
| GID7400704 | 9 | PBW343/TONI//ELVIRA/7/CNO79//PF70354/MUS/3/PASTOR/4/BAV92/5/FRET2/KUKUNA//FRET2/6/MILAN/KAUZ//PRINIA/3/BAV92 |
| GID7400738 | 9 | WHEAR/SOKOLL/8/BOW/VEE/5/ND/VG9144//KAL/BB/3/YACO/4/CHIL/6/CASKOR/3/CROC_1/AE.SQUARROSA (224)//OPATA/7/PASTOR//MILAN/KAUZ/3/BAV92 |
| GID7400748 | 11 | SUP152/6/OASIS/5*BORL95/5/CNDO/R143//ENTE/MEXI75/3/AE.SQ/4/2*OCI |
| GID7400769 | 9 | NELOKI//SOKOLL/EXCALIBUR |
| GID7400771 | 9 | NELOKI//SOKOLL/EXCALIBUR |
| GID7400794 | 11 | LIVINGSTON/6/2*MTRWA92.161/PRINIA/5/SERI*3//RL6010/4*YR/3/PASTOR/4/BAV92 |

**Table S2**. Statistical analysis of spot blotch incidence in 139 wheat genotypes evaluated at two locations in three environments.

| **Description** | ***Env1*** | ***Env2*** | ***Env3*** |
| --- | --- | --- | --- |
| Mean | 21.88 | 21.78 | 7.72 |
| Standard Error | 0.48 | 0.49 | 0.54 |
| Median | 20.52 | 20.43 | 5.91 |
| Mode | 18.72 | 18.54 | 3.06 |
| Standard Deviation | 5.69 | 5.83 | 6.37 |
| Sample Variance | 32.43 | 34.04 | 40.59 |
| Kurtosis | -0.04 | 0.02 | 5.93 |
| Skewness | 0.64 | 0.60 | 2.31 |
| Range | 13.33 | 26.49 | 34.64 |
| Minimum | 13.33 | 12.86 | 2.65 |
| Maximum | 38.52 | 39.35 | 37.29 |
| Count | 139.00 | 139.00 | 139.00 |
| Largest (1) | 38.52 | 39.35 | 37.29 |
| Smallest (1) | 13.33 | 12.86 | 2.65 |
| Confidence Level (95.0%) | 0.96 | 0.98 | 1.07 |

| T**able S3.** Monthly average, minimum and maximum temperatures at BISA, Pusa in India | | | | | | | | | | | | |
| --- | --- | --- | --- | --- | --- | --- | --- | --- | --- | --- | --- | --- |
|  | Oct | Nov | Dec | Jan | Feb | Mar | Apr | May | Jun | Jul | Aug | Sep |
| **Pusa, Bihar, India** | | |  |  |  |  |  |  |  |  |  |  |
| Avg. Temp (°C) | 26.9 | 22.0 | 18.1 | 16.8 | 19.2 | 24.6 | 28.8 | 31.4 | 30.8 | 29.4 | 29.3 | 28.9 |
| Min. Temp (°C) | 22.4 | 15.3 | 11.0 | 10.1 | 12.2 | 17 | 22.1 | 25.3 | 26.4 | 26.3 | 26.4 | 25.8 |
| Max. Temp (°C) | 31.5 | 28.8 | 25.2 | 23.6 | 26.3 | 32.3 | 35.6 | 37.5 | 35.3 | 32.6 | 32.2 | 32.1 |
| **BARI, Jamalpur, Bangladesh** | | |  |  |  |  |  |  |  |  |  |  |
| Avg. Temp (°C) | 27 | 23.2 | 19.8 | 18.4 | 20.8 | 25.2 | 28.4 | 28.4 | 28.1 | 28.4 | 28.5 | 28.4 |
| Min. Temp (°C) | 23.2 | 17.8 | 13.7 | 12.0 | 13.8 | 18.3 | 22.4 | 24.0 | 25.0 | 25.7 | 25.9 | 25.5 |
| Max. Temp (°C) | 30.8 | 28.6 | 26.0 | 24.9 | 27.9 | 32.1 | 34.4 | 32.8 | 31.3 | 31.1 | 31.1 | 31.3 |
| Source: https://en.climate-data.org | | | | | | | | | | | | |
